# Visual and optical quality of enhanced intermediate monofocal versus standard monofocal intraocular lens

**DOI:** 10.1007/s00417-022-05700-y

**Published:** 2022-05-27

**Authors:** Nuria Garzón, Francisco Poyales, César Albarrán-Diego, Laura Rico-del-Viejo, Lidia Pérez-Sanz, María García-Montero

**Affiliations:** 1 Miranza Group, Madrid, C/Galileo, 104, 28003 Madrid, Spain; 2 Optometry and Vision Department, Faculty of Optics and Optometry, Complutense University of Madrid, 28037 Madrid, Spain; 3 Optics, Optometry and Vision Science Department, Faculty of Physics, University of Valencia, Dr Moliner 50, 46100 Burjassot, Spain; 4 Clínica Oftalmológica Dr Gonzalo Muñoz, C/Marqués de Sotelo 5, 46002 Valencia, Spain

**Keywords:** Cataract surgery, Monofocal intraocular lens, Monofocal with enhanced intermediate vision lens, Refraction

## Abstract

**Purpose:**

Intraocular lens designs are constantly evolving, trying to obtain more spectacle independence after cataract surgery. This advantage can be linked to some disadvantages, such as optical quality decrease. For that reason, it is important to assess, not only the amount of vision provided but also the quality of vision once they are implanted. The purpose of the present work was to compare the visual performance between two monofocal intraocular models: a standard model and a monofocal with enhanced intermediate vision lens.

**Methods:**

Prospective, randomized, comparative study. Sixty adult subjects scheduled to undergo bilateral cataract surgery and IOL implantation were randomized to receive one of the two IOLs in both eyes at Miranza IOA, Madrid, Spain (group A: monofocal with enhanced intermediate vision lens and group B: standard monofocal lens). Monocular outcomes (right eyes) determined 1 and 3 months postoperatively were photopic corrected distance visual acuity (CDVA), uncorrected distance visual acuity (UDVA), perceived halo, corrected intermediate-distance contrast sensitivity, and higher-order aberrations. The impact of the new IOL in the postoperative management with autorefraction devices was also evaluated.

**Results:**

No differences were found in CDVA between the two groups. Significant differences were detected between the two lenses evaluated in both total HOA (*p* = 0.028) and internal HOA (*p* = 0.037). Contrast sensitivity and halometry results obtained at 1 month were similar across the two IOL groups.

**Conclusion:**

In patients undergoing cataract surgery, monofocal with enhanced intermediate vision IOL offered similar distance performance and contrast sensitivity along with perceived HOA and halos compared with the standard monofocal IOLs tested.


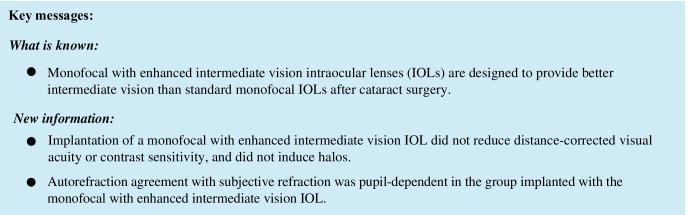


## Introduction

Intraocular lenses (IOLs) used to replace the natural lens for refractive lens exchange or cataracts have evolved considerable over the years. There is currently a wide array of IOLs available spanning from advanced or enhanced monofocal designs to multifocals or extended depth-of-focus lenses offering good vision at more than just one distance, but with some optical quality sacrifice. [1, 2]

One of the newer IOL models is the monofocal with enhanced intermediate vision TECNIS® Eyhance model ICB00 (Johnson & Johnson Vision, USA). While this IOL can be classified as a monofocal lens, it offers a steady gradual change in lens power from the periphery towards the centre. This design creates an anterior IOL surface that improves intermediate vision (66 cm) while maintaining the good distance vision quality offered by other aspheric monofocal IOLs and also minimizing the dysphotopsia phenomena that characterize distant-dominant multifocal IOLs.^3^

In the present study, we did not compare intermediate visual acuity between standard and enhanced monofocal IOLs, since our goal was just to examine whether the addition of improved intermediate vision with the new enhanced monofocal IOLs came at any sacrifice to the quality of the distance vision when compared to the more conventional monofocal IOLs. For this purpose, we compared distance vision outcomes and other factors such as photic phenomenon affecting vision quality between the TECNIS® Eyhance model ICB00 IOL (Johnson & Johnson Vision, USA) and the standard monofocal design TECNIS® One model ZCB00 (Johnson & Johnson Vision, USA) in cataract patients not seeking for spectacle independence. Furthermore, the impact of this new IOL design, aimed to improve intermediate vision, in the postoperative management with autorefraction devices was evaluated too.

## Materials and methods

This was a prospective randomized study. Both the patients and the optometrist performing postsurgery testing were blind to the type of IOL implanted, whereas surgeon was aware to the type of IOL implanted in each patient. According to our sample size calculation, we enrolled 60 subjects. The sample size was calculated based on the results published by Bellucci et al., related to visual acuity, to recognize statistically significant differences with a standard deviation assumed to be 0.08, accepting an alpha risk of 0.05 and a beta risk of 0.2.^4^ These participants were divided into two groups of 30 patients randomized to undergo cataract surgery with the bilateral implant of the IOLs TECNIS® Eyhance ICB00 (group A) or TECNIS® One ZCB00 (group B). All patients enrolled in the study underwent bilateral symmetric IOL implantation, but only right eyes were measured for monocular variables.^5^

The study protocol adhered to the tenets of the declaration of Helsinki and was approved by the Ethic Committee of the Hospital Clínico San Carlos, Madrid, Spain. Before enrollment, written informed consent was obtained from each patient.

Inclusion criteria for this study were as follows: age > 50 years, potential visual acuity > 0.2 LogMAR, corneal astigmatism with the rule < 1.50 D and against the rule or oblique < 1.00 D, no previous surgery or ocular trauma, and no disorders that could affect surgery such as pseudoexfoliative syndrome or comorbidities that could affect the final outcome.

All patients were subjected to a full ophthalmologic examination before and after surgery. Presurgery tests were manifest refraction and monocular distance visual acuity, corneal topography, slit-lamp biomicroscopy, tonometry, pupillometry, fundus exam, and macular optical coherence tomography (OCT). IOL power was calculated through optical coherence interferometry (IOL Master 700; Carl Zeiss Meditec., Jena, Germany). We used the Barrett Universal II formula for IOL power calculations, with an optimized constant of 2.04.

The surgical procedure was similar for both lenses. All surgeries were carried out by the same surgeon (FP) under topical anesthesia. Anterior capsulotomy and nuclear fragmentation were performed with a femtosecond laser (CATALYS Precision System, Johnson & Johnson, Santa Ana, CA). A 2.2 mm temporal corneal incision and a paracentesis were made with a surgical knife, and for lens phacoemulsification a commercial microsurgical system (Centurion Vision System; Alcon Laboratories, Inc., Fort Worth, TX) was employed. The chosen IOL was then implanted into the capsular bag with a single-use injection system. All surgeries were supported by the computer-assisted cataract surgery system (CALLISTO Eye from Zeiss’ Cataract Suite Markerless; Carl Zeiss, Jena, Germany).

The postsurgery tests for this study were conducted 1 and 3 months after surgery. The measurements made in these two follow-up visits were objective refraction (ObjRx) under both photopic and mesopic conditions, uncorrected (UDVA) and corrected (CDVA) visual acuity at a distance of 4 m (Clinical Trial Suite, M&S Technologies, USA), halo, photopic contrast sensitivity and total, corneal and internal higher order (up to sixth) aberrations.

Halo was measured using the free-access software Halo v1.0 (Laboratorio de Ciencias de la Visión y Aplicaciones, Universidad de Granada, Spain).^6,7^ This test detects and quantifies the halo perceived by a subject under conditions of low light. In this test, the subject identifies peripheral light stimuli that randomly arise around a high luminance central point on a dark background. Halo is measured as the discrimination index, which is related to the radius of the area where the peripheral stimuli cannot be detected by the subject and ranges from 0 to 1.^7^ The higher this index, the lesser the halo effect. The test screen is placed at 4 m, and the test is conducted monocularly in scotopic luminance conditions.

Contrast sensitivity was measured at a distance of 2.5 m with best distance correction using the test CSV-1000 (Vector Vision Inc., Greenville, OH, USA). The CSV-1000 is a translucent retro-illuminated chart that consists of a series of circular achromatic sine-wave patches. The chart is divided into 4 sinewave grating stimuli (spatial frequencies of 3, 6, 12, and 18 cycles/degree) and 8 levels of contrast.

Higher-order aberrations (HOA) were measured using the OPD III Scan system (OPD-Scan III, Nidek Co., Ltd.) through the root mean square (RMS). This device is a wavefront topography aberrometry system that relies on the principle of scanning-slit retinoscopy whereby the retina is scanned with an infrared slit beam. RMS values were obtained of total, corneal and internal HOA for a mesopic pupil.

The objective refraction (ObjRx) was obtained with two methods, as described elsewhere.^8^ The first one was by using the KR8800 autorefractor (Topcon Inc, Tokyo, Japan). The second method to measure ObjRx was with the Nidek OPD-Scan III (Nidek Technologies, Gamagori, Japan), a device combining a wavefront aberrometer, an autorefractor, and a pupillometer. This device provides objective refraction values both under photopic and mesopic lighting conditions. Pupils, under photopic and mesopic conditions, were measured with OPD III Scan (Nidek Technologies, Gamagori, Japan). The values measured were the OPD-C (automated refraction measured with the aberrometer OPD in the central pupil/photopic conditions), and OPD-M (automated refraction measured with the aberrometer OPD under mesopic conditions).

### Intraocular lenses

The IOLs compared were TECNIS® Eyhance ICB00 (Johnson & Johnson, CA, USA), and TECNIS® One ZCB00 (Johnson & Johnson, CA, USA). Both lenses are aspheric, acrylic hydrophobic, one-piece lenses.

The TECNIS® Eyhance model ICB00 features a smooth continuous power change from the centre towards the lens periphery. This feature seems to increase depth of focus, thus mitigating the effects of presbyopia and delivering sharp vision at different distances. Both TECNIS® models have a modified anterior surface with an asphericity of − 0.27 μm across their entire optic zone of 6 mm.

The difference between the two TECNIS® IOLs is that model TECNIS® One model ZCB00 is exclusively monofocal, while the TECNIS® Eyhance model ICB00 is monofocal with enhanced intermediate function. Both lenses have a 360 degrees continuous posterior squared margin. Their Abbe number is 55 and refraction index is 1.47.

### Statistical analysis

Data analysis was performed using the software package SPSS for Windows version 22.0 (IBM, Armonk, NY, USA). The sample size was calculated based on the results published by Bellucci et al.^4^ related to visual acuity, to recognize statistically significant differences with a standard deviation assumed to be 0.08, accepting an alpha risk of 0.05 and a beta risk of 0.2. Rx and ObjRx values obtained in clinical spherocylindrical notation, were converted into power-vector notation for comparison purposes.^9^

Descriptive data for the IOLs are provided as means and their standard deviations (SD). The normality of the data was checked using the Shapiro–Wilk test. The Student *t* test for unpaired data or Mann–Whitney test was used for comparisons between the two groups. *P* value less than 0.05 was considered statistically significant.

## Results

Preoperative data for the two groups of patients receiving both IOL models are provided in Table 1. There were no statistically significant differences between the two patient groups (those implanted with the TECNIS® Eyhance model ICB00 and with the TECNIS® One model ZCB00) for any of the parameters, except for age, whose *p* was 0.002. No significant intra-op or post-op complications were noted in either arm.

**Table 1:** Preoperative patient data for the IOLs examined in this study

	Group A (ICB-IOL)	Group B (ZCB-IOL)
Gender (M/F) %	22.4/77.6	37.1/62.9
Age (years)	75.87 ± 5.97 (61 to 87)	70.62 ± 8.11 (56 to 84)
Spherical equivalent (D)	− 0.49 ± 2.84 (+ 4.00 to − 6.75)	− 0.75 ± 3.09 (+ 4.25 to − 8.75)
CDVA (LogMAR)	0.19 ± 0.15 (0.00 to 0.70)	0.20 ± 0.19 (0.00 to 1.00)
Photopic pupil size (mm)	3.21 ± 0.56 (1.50 to 4.67)	3.04 ± 0.52 (2.23 to 4.29)
Mesopic pupil size (mm)	4.32 ± 0.84 (2.00 to 6.49)	4.11 ± 0.68 (2.71 to 5.37)
AXL (mm)	23.30 ± 1.00 (21.62 to 26.60)	23.77 ± 1.42 (21.58 to 26.65)
ACD (mm)	3.04 ± 0.36 (2.21 to 3.92)	3.10 ± 0.31 (2.59 to 3.72)
Kmax (D)	44.61 ± 1.42 (41.28 to 47.81)	44.48 ± 1.60 (41.26 to 49.05)
IOL power (D)	21.96 ± 2.23 (14.50 to 26.00)	20.72 ± 4.16 (14.00 to 27.50)

Group A (ICB-IOL) = enhanced monofocal lens TECNIS® Eyhance model ICB00; group B (ZCB-IOL) = standard monofocal lens TECNIS.® One model ZCB00

*CDVA* corrected distance visual acuity, *AXL* axial length, *ACD* anterior chamber depth, *Kmax* maximum corneal curvature, *D* diopters, *mm* millimeters, *LogMAR* logarithm of the minimum angle of resolution

No differences between both IOL groups were found nor in UDVA neither in CDVA outcomes (Table 2) and no differences were observed in the halometry results obtained 1 month post-surgery (Table 2).

**Table 2:** Postoperative patient data for the IOLs examined in this study

		Group A (ICB-IOL)	Group B (ZCB-IOL)
UDVA (LogMAR)		0.16 ± 0.24 (− 0.06 to 1.0)	0.13 ± 0.23 (− 0.06 to 1.0)
CDVA (LogMAR)		0.02 ± 0.05 (− 0.10 to 0.15)	0.01 ± 0.04 (− 0.10 to 0.15)
Halo (discrimination index)		0.819 ± 0.117 (0.345 to 0.981)	0.828 ± 0.121 (0.454 to 0.981)
Contrast sensitivity (cpd)	3 cpd	1.56 ± 0.23 1.00 to 2.07)	1.56 ± 0.18 (1.17 to 1.785)
	6 cpd	1.61 ± 0.27 (0.90 to 1.99)	1.73 ± 0.25 (0.90 to 2.14)
	12 cpd	1.15 ± 0.31 (0.60 to 1.69)	1.25 ± 0.30 (0.60 to 1.69)
	18 cpd	0.67 ± 0.28 (0.17 to 1.39)	0.82 ± 0.34 (0.17 to 1.55)
HOA RMS (µm)	Total	0.39 ± 0.26 (0.11 to 1.3)	0.26 ± 0.13 (0.07 to 0.70)
	Corneal	0.28 ± 0.15 (0.07 to 0.76)	0.33 ± 0.63 (0.06 to 2.89)
	Internal	0.39 ± 0.28 (0.13 to 1.30)	0.27 ± 0.11 (0.13 to 0.65)

Group A (ICB-IOL) = enhanced monofocal lens TECNIS® Eyhance model ICB00; group B (ZCB-IOL) = standard monofocal lens TECNIS.® One model ZCB00

*UDVA* uncorrected distance visual acuity, *CDVA* corrected distance visual acuity, *RMS* root mean square, *HOA* higher order aberrations, *D* diopters, *cpd* cycles per degree, *µm* microns, *LogMAR* logarithm of the minimum angle of resolution

**Fig. 1:**
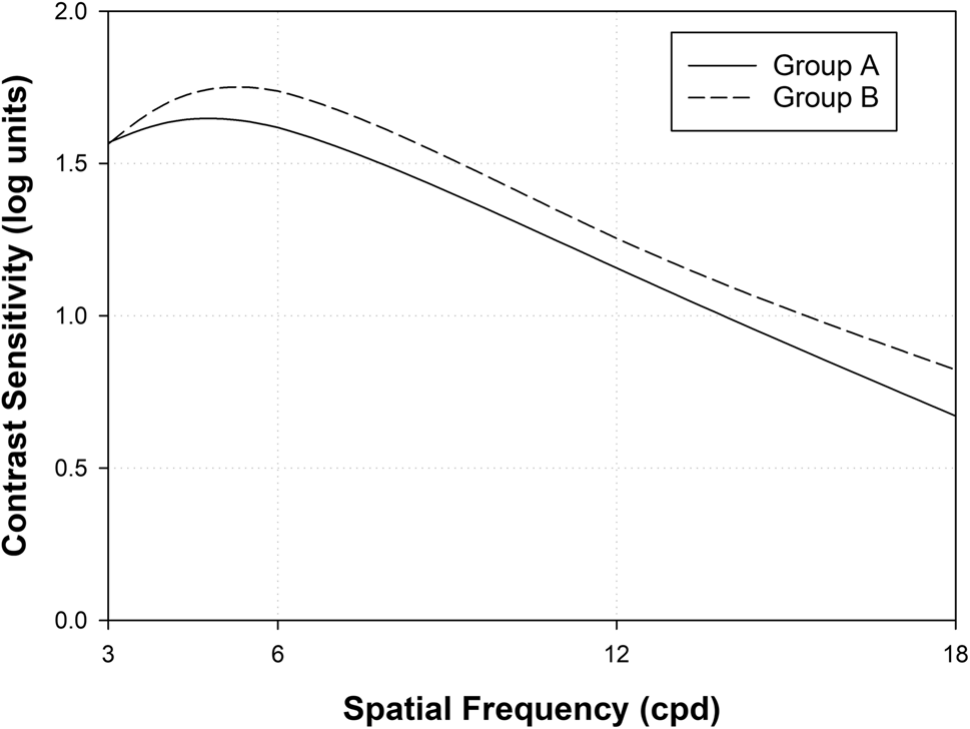
Contrast sensitivities recorded in the CSV-1000 test at different spatial frequencies (3, 6, 12, 18 cpd) for the intraocular lenses implanted. Solid line = group A group is the enhanced monofocal lens TECNIS® Eyhance, dashed line = group B is the standard monofocal lens TECNIS.® One

Contrast sensitivity values for both IOL groups acquired in photopic conditions are illustrated in Fig. 1 and descriptive contrast sensitivity data in logarithmic units are provided in Table 2. No differences emerged between the two groups for any of the examined spatial frequencies (3, 6, 12, and 18 cycles/degree).

In Table 2, we also provide descriptive data for higher order aberrations obtained in the two IOL models. Significant differences were detected between groups in both total HOA (*p* = 0.028) and internal HOA (*p* = 0.037), but no differences were found for corneal HOA (*p* = 0.693).

About the objective and subjective refraction, Table 3 shows a summary of the surgical outcomes in terms of refraction for both groups. The average refractive result was close to emmetropia, with a mean spherical equivalent of − 0.13 D and − 0.32 D for group A and group B, respectively.

**Table 3:** Descriptive statistics obtained after surgery for refraction and visual acuity in both groups

		Mean ± SD		Range	
		Group A (ICB-IOL)	Group B (ZCB-IOL)	Group A (ICB-IOL)	Group B (ZCB-IOL)
Refraction (D)	M	− 0.32 ± 0.87	− 0.13 ± 0.74	− 2.75 to + 0.75	− 2.88 to + 0.50
	J_0_	− 0.11 ± 0.19	− 0.16 ± 0.21	− 0.50 to + 0.13	− 0.70 to + 0.25
	J_45_	0.00 ± 0.17	0.04 ± 0.13	− 0.25 to + 0.38	− 0.22 to + 0.32
	Sph	− 0.12 ± 0.86	0.07 ± 0.69	− 2.50 to + 0.75	− 2.50 to + 0.50
	Cyl	− 0.39 ± 0.38	− 0.41 ± 0.43	− 1.00 to 0.00	− 1.50 to 0.00

Group A (ICB-IOL) = enhanced monofocal lens TECNIS® Eyhance model ICB00; group B (ZCB-IOL) = standard monofocal lens TECNIS.® One model ZCB00

*M* spherical equivalent, *J*_*0*_ and *J*_*45*_ astigmatic vector components, *Sph* sphere, *Cyl* cylinder, *UDVA* uncorrected distance visual acuity, *DCVA* distance corrected visual acuity, *SD* standard deviation, *D* diopters

Table 4 shows the mean ± SD values for objective results obtained in both groups.

**Table 4:** Descriptive statistics (average ± standard deviation) for the objective refractions obtained with all the evaluated methods, for both groups (expressed in diopters)

		AR	OPDC	OPDM
Sph	Group A (ICB-IOL)	− 0.14 ± 0.80	− 0.24 ± 0.80	0.04 ± 0.82
	Group B (ZCB-IOL)	− 0.18 ± 0.92	− 0.42 ± 0.78	− 0.23 ± 0.74
Cyl	Group A (ICB-IOL)	− 0.51 ± 0.48	− 0.57 ± 0.47	− 0.41 ± 0.67
	Group B (ZCB-IOL)	− 0.58 ± 0.59	− 0.53 ± 0.41	− 0.55 ± 0.64
M	Group A (ICB-IOL)	− 0.39 ± 0.77	− 0.53 ± 0.79	− 0.16 ± 0.89
	Group B (ZCB-IOL)	− 0.47 ± 0.93	− 0.69 ± 0.82	− 0.51 ± 0.87
J_0_	Group A (ICB-IOL)	− 0.18 ± 0.25	− 0.18 ± 0.28	− 0.06 ± 0.26
	Group B (ZCB-IOL)	− 0.16 ± 0.29	− 0.13 ± 0.21	− 0.12 ± 0.32
J_45_	Group A (ICB-IOL)	0.00 ± 0.17	0.02 ± 0.17	0.07 ± 0.27
	Group B (ZCB-IOL)	0.02 ± 0.25	0.02 ± 0.23	0.00 ± 0.25

Group A (ICB-IOL) = enhanced monofocal lens TECNIS® Eyhance model ICB00; group B (ZCB-IOL) = standard monofocal lens TECNIS.® One model ZCB00

*Sph* sphere, *Cyl* cylinder, *M* spherical equivalent, *J*_*0*_ vertical Jackson cross-cylinder, axes at 180° and 90°, *J*_*45*_ oblique Jackson cross-cylinder, axes at 45° and 135°, *AR* autorefraction, *OPDC* autorefraction measured with the 3-dimension wavefront topography aberrometer system in the central pupil/photopic conditions, *OPDM* autorefraction measured with the 3-dimension wavefront topography aberrometer system under mesopic conditions

**Fig. 2:**
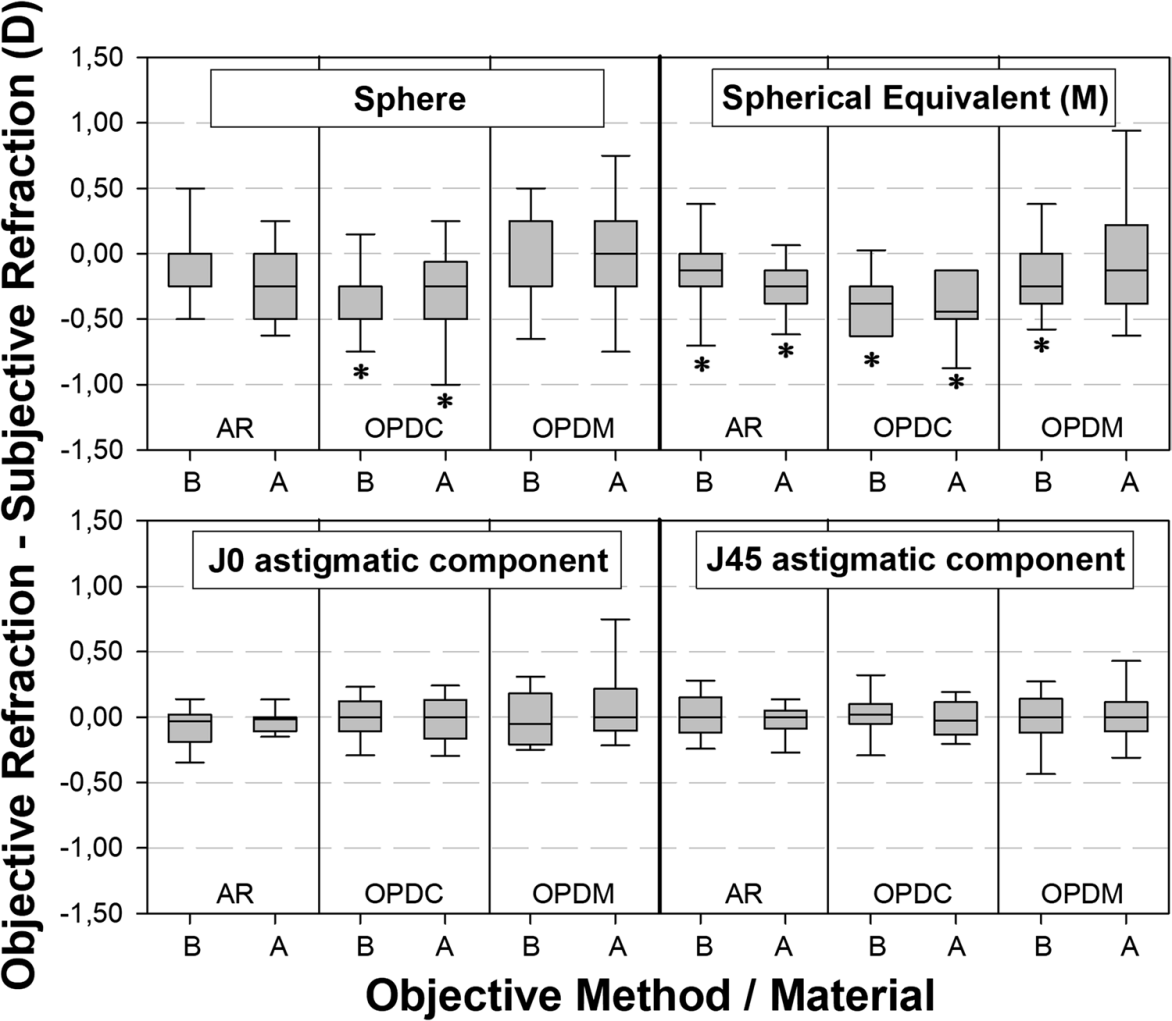
Objective–subjective refraction difference of Sph (sphere), M (spherical equivalent), and J_0_ and J_45_ (vector components of astigmatism) versus the objective refraction method in in both the group implanted with the standard monofocal lens TECNIS.® One model ZCB00 (B) and the group with enhanced monofocal lens TECNIS® Eyhance model ICB00 (A). There are three objective refraction scenarios under assessment (AR = autorefraction; OPD-C = autorefraction measured with the 3-dimension wavefront topography aberrometer system in the central pupil/photopic conditions; OPD-M = autorefraction measured with the 3-dimension wavefront topography aberrometer system under mesopic conditions). The asterisks (*) indicates statistically significant differences

Figure 2 shows a boxplot illustrating the differences between Rx outcomes and each of the 3 ObjRx measuring approaches under evaluation, for sphere (S), spherical equivalent (M) and astigmatism components (J_0_ and J_45_) in both the group implanted with the enhanced monofocal lens TECNIS® Eyhance model ICB00 and the group implanted with the standard monofocal lens TECNIS® Tecnis One model ZCB00. For the group A, the Friedman repeated measures analysis of variance on ranks revealed statistically significant differences among refraction measurement methods for Sphere (*p* < 0.001) just for the comparison Rx vs OPD-C (Tukey, *p* = 0.009), but not for Rx vs AR (Tukey *p* = 0.05), or Rx vs OPD-M (Tukey, *p* = 0.862). For the M component in the ICB-IOL group, the Friedman repeated measures analysis of variance on ranks revealed differences among methods (*p* < 0.001), located by the Tukey test in the comparisons Rx vs AR (*p* = 0.001), and Rx vs OPD-C (*p* < 0.001), but not in the comparison Rx vs OPD-M (*p* = 0.536). No differences were found in the ICB-IOL group by the Friedman repeated measures analysis of variance on ranks for J0 (*p* = 0.558) and J45 (*p* = 0.924). For the group B, the two-way repeated measures ANOVA revealed statistically significant differences among refraction measurement methods (*p* < 0.001), and also interactions between refractive component and measurement method (*p* < 0.001). Holm–Sidak pairwise testing located the differences in sphere just for the comparison Rx vs OPD-C (*p* < 0.001), but no differences were found for the comparison Rx vs AR (*p* = 0.504) or Rx vs OPD-M (*p* = 0.226). Differences for spherical equivalent (M) were found for the comparison of Rx vs all the objective methods: Rx vs AR (*p* = 0.023), Rx vs OPD-C (*p* < 0.001), and Rx vs OPD-M (*p* = 0.009). No differences in the ZCB-IOL group were found for the J_0_ and J_45_ astigmatic components (*p* > 0.05 in all pairwise comparisons).

**Fig. 3:**
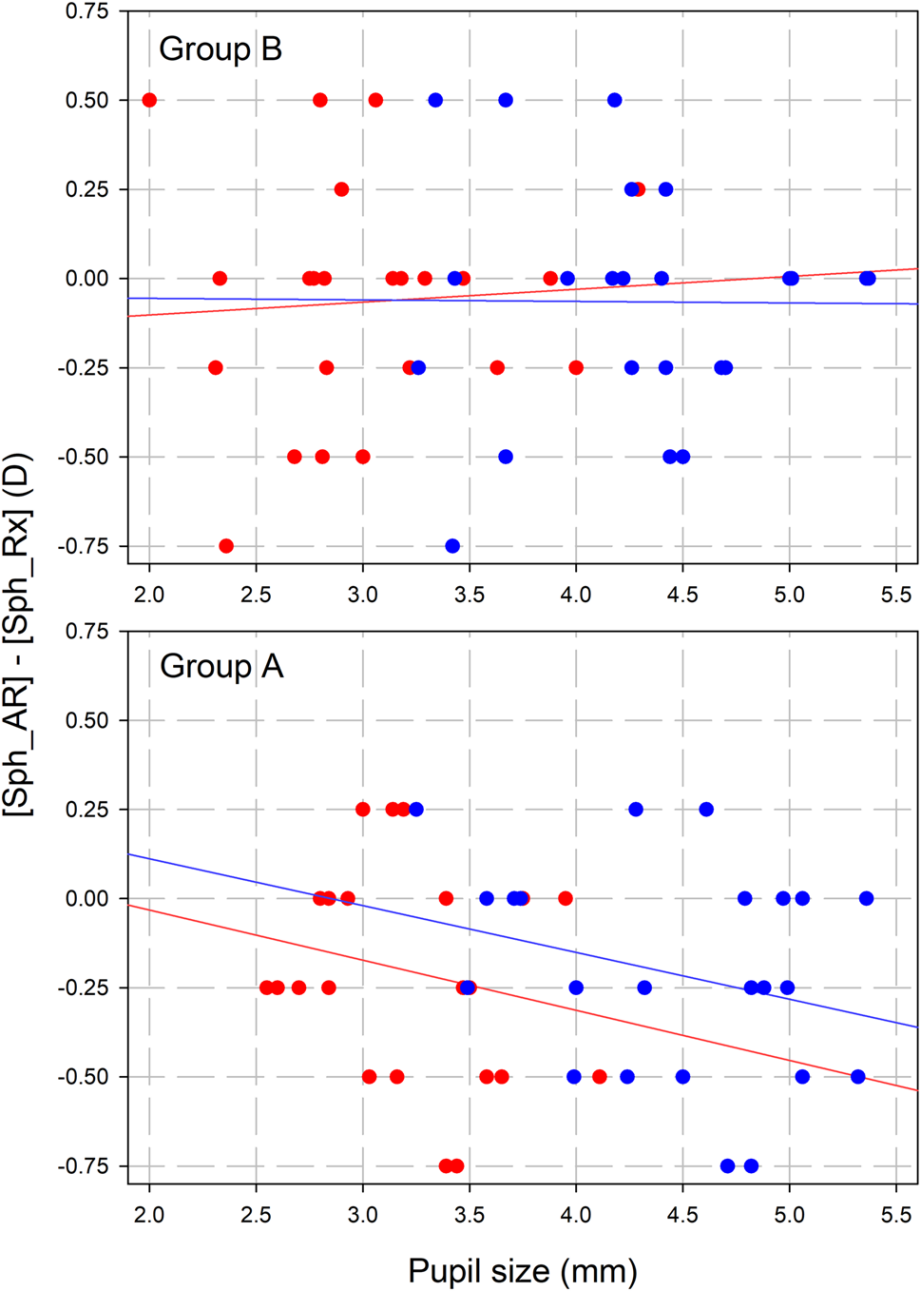
Differences between subjective refraction (Rx) and automated refraction (AR) related to pupil size in both lens. Group B (upper part) is the standard monofocal lens TECNIS.® One model ZCB00 and Group A (lower part) is the enhanced monofocal lens TECNIS® Eyhance model ICB00. Red dots represent photopic data and blue dots represent mesopic data. Solid lines represent the linear regression of both photopic (red line) and mesopic (blue line) data. Sph = sphere, AR = automated refraction, Rx = subjective refraction, D = diopters

Figure 3 shows the differences in sphere between Rx and AR related to pupil size, both for photopic (red dots) and mesopic (blue dots) lighting conditions. The upper part of Fig. 3 shows the ZCB-IOL data and the lower part shows the ICB-IOL data. Solid lines represent the linear regression of scatter data.

## Discussion

Intraocular lens designs are constantly evolving. One of the last marketed models is the monofocal with enhanced intermediate vision TECNIS® Eyhance model ICB00. Several studies have confirmed that this lens delivers improved intermediate vision than that observed for monofocal lenses.^10,11^

In a laboratory study of IOL surface profiles based on contact profilometry, Tognetto et al. described the TECNIS® Eyhance model ICB00 lens as monofocal lens based on a high order aspheric optic offering improved intermediate vision with just minimal surface geometry differences in its central design compared to standard monofocal IOLs (Sensar AAB00, Tecnis ZCB00, Mini 4 Ready).^12^ Based on the notion that this subtle difference in the lens center could affect the optical and visual quality perceived by the patient, we compared these factors with those observed for a standard purely monofocal IOL.

Our corrected distance visual acuity data (0.02 ± 0.05 for ICB-IOL group and 0.01 ± 0.04 for ZCB-IOL group) are in line with those reported by Mencucci et al. ^10^ for both TECNIS® models: 0.02 ± 0.04 for TECNIS® Eyhance model ICB00 and 0.03 ± 0.05 for TECNIS® One model ZCB00. As in our study, these authors detected no significant differences in CDVA between their two groups of patients. Auffarth et al.^11^ also compared these two IOL models reporting similar CDVA outcomes (− 0.02 ± 0.01 and − 0.06 ± 0.01 for ICB-IOL and ZCB-IOL models, respectively) with no differences between IOL models. Judging by the results observed in our study and in studies by Mencucci et al.^10^ and Auffarth et al.^11^, it seems that distance visual outcome differences between both evaluated IOLs are neither clinically relevant nor statistically significant.

Contrast sensitivity outcomes for the TECNIS® lenses were also examined by Mencucci et al.^10^ Neither did these authors detect significant differences between the two models although at all spatial frequencies examined, contrast sensitivities for TECNIS® One model ZCB00 were the same or slightly better than for TECNIS® Eyhance model ICB00, with higher values detected in both cases at a frequency of 6 cpd. These data are consistent with those recorded here for these two IOL models. So, it seems that the variation conducted in the anterior surface of the TECNIS® Eyhance model ICB00 looking for a better intermediate vision did not translate into a worsening of far distance optical quality.

Photic phenomena provoked by the TECNIS® Eyhance model ICB00 lens were examined by several authors^13,14^ in a laboratory study. Vegat et al.^13^ noted halos around a pinhole image that could be hardly observed for IOL-pupil sizes up to 3 mm, but became apparent for larger IOL-pupil sizes of 4 and 5 mm. Their conclusion was that on an optical bench the new design of the TECNIS® Eyhance model ICB00 is more likely to induce a similar level of photic phenomenon, if any, as the TECNIS® One model ZCB00. Our findings in dim light conditions are in agreement with these conclusions. The TECNIS® Eyhance model ICB00, despite its central higher-order aspherical design, was perceived by patients to induce similar halos as the standard monofocals.

Since the new TECNIS® Eyhance ICB00 has a modified anterior optical surface inducing spherical aberration in order to enhance intermediate vision, it could be expected a higher value of RMS for HOA in this group compared with the TECNIS® One ZCB00 group. This supposition is in agreement with our results: higher values of total (cornea + IOL) and internal (IOL) HOA were found in the group implanted with TECNIS® Eyhance ICB00 given its higher value of induced spherical aberration, without differences between both groups in corneal aberrations. This result is also in agreement with Vega et al.^13^ who compared in an optical bench the same two lenses tested in the present work, and found spherical aberration amounts 3.7 times larger for the TECNIS® Eyhance ICB00 compared to the TECNIS® One ZCB00 for pupils smaller than 3.5 mm. But despite those statistically significant differences in internal HOA found in our study between TECNIS® Eyhance ICB00 (0.39 ± 0.28 μm) compared with the TECNIS® One ZCB00 (0.27 ± 0.11 μm), the rest of results (same visual acuity and same contrast sensitivity) suggests that those differences are clinically irrelevant in terms of the possible impact in far vision visual quality. That is, the amount of HOA induced by the new TECNIS® Eyhance ICB00 in order to enhance intermediate vision, which, by definition, would degrade optical quality, do not produce a relevant degradation in perceived far distance visual quality, as demonstrated by visual acuity, haloes and contrast sensitivity values. This result points the importance of optical, but also neural-processing factors involved in visual perception, with the second ones alleviating the possible impact of the first ones in the final visual perception process. We can think that the amount of spherical aberration induced by this lens has been optimized to enhance intermediate vision without having clinically relevant impact in far vision, so it could also be thought that it is possible to improve the already enhanced intermediate vision (even achieving near vision) by inducing higher values of spherical aberration, but probably with a clinical impact in far vision visual quality.

Regarding postoperative management in patients implanted with both evaluated IOL designs, the wavefront-based device used to obtain ObjRx (OPD-Scan III, Nidek Technologies, Gamagori, Japan) did not show superior agreement with Rx than classic AR measurement. Agreement was superior for cylinder than for sphere, in both IOL groups. Sphere showed similar average agreement for both model ICB00 and ZCB00 implanted eyes, with worst agreement results obtained measuring with the OPD-Scan III under photopic conditions. Once AR showed noninferiority to OPD-Scan for ObjRx measurement, spherical agreement between AR and Rx as a function of pupil size was analyzed. As can be seen in Fig. 3, the ZCB-IOL group showed a non-pupil-dependent result, whereas ICB-ICL group showed different agreement between AR and Rx depending on pupil size, in such a way that the biggest is the pupil, the more different (more negative) is the AR result comparing to the actual Rx. So, with a bigger pupil, it can be expected a more negative value of AR in patients implanted with ICB-IOL. This fact must be taken into account for the clinical determination of Rx from ObjRx values.

Although in our study the age distribution in both groups is significantly different, we do not think that this could be a real source of bias, since the subjects were randomly assigned to the study groups. Age could influence visual results through pupil size, in such a way that older subjects with more miotic pupils could be expected to have better vision given the greater depth of field and the lesser effect of aberrations. But in our study, no significant differences were found in pupil size between the groups.

## Conclusion

The findings of our study indicate that the modification of the anterior surface of the TECNIS® One model ZCB00 IOL to get the TECNIS® Eyhance model ICB00 IOL designed to offer the patient better visual acuity and spectacle-independence at intermediate distance, had no negative impact on the far distance quality of vision both measured and perceived by the patient, in terms of VA, contrast sensitivity, halos or aberrations. A possible pupil dependence behavior can be expected when measuring ObjRx in eyes implanted with TECNIS® Eyhance model ICB00 IOL.
